# Anti-inflammatory Action of Statins in Cardiovascular Disease: the Role of Inflammasome and Toll-Like Receptor Pathways

**DOI:** 10.1007/s12016-020-08791-9

**Published:** 2020-05-06

**Authors:** Khadijeh Koushki, Sanaz Keshavarz Shahbaz, Kazem Mashayekhi, Mahvash Sadeghi, Zeinab Deris Zayeri, Mahdieh Yousefi Taba, Maciej Banach, Khalid Al-Rasadi, Thomas P. Johnston, Amirhossein Sahebkar

**Affiliations:** 1 Department of Immunology, Faculty of medicine, Mashhad University of Medical Sciences, Mashhad, Iran; 2 Golestan Hospital Clinical Research Development Unit, Ahvaz Jundishapur University of Medical Sciences, Ahvaz, Iran; 3 Mashhad University of Medical Science, Mashhad, Iran; 4 Department of Hypertension, WAM University Hospital in Lodz, Medical University of Lodz, Zeromskiego 113, Lodz, Poland; 5 Polish Mother’s Memorial Hospital Research Institute (PMMHRI), Lodz, Poland; 6 Medical Research Centre, Sultan Qaboos University, Muscat, Oman; 7 Department of Biochemistry, College of Medicine and Health Sciences, Sultan Qaboos University, Muscat, Oman; 8 Division of Pharmacology and Pharmaceutical Sciences, School of Pharmacy, University of Missouri-Kansas City, Kansas City, MO USA; 9 Halal Research Center of IRI, FDA, Tehran, Iran; 10 Biotechnology Research Center, Pharmaceutical Technology Institute, Mashhad University of Medical Sciences, Mashhad, Iran; 11 Neurogenic Inflammation Research Center, Mashhad University of Medical Sciences, Mashhad, Iran

**Keywords:** Statins, NLRP3, Inflammasome, TLR4, TLR2, Cardiovascular disease, Atherosclerosis

## Abstract

Atherosclerosis is one type of cardiovascular disease (CVD) in which activation of the NLRP3 inflammasome and toll-like receptor (TLR) pathways is implicated. One of the most effective treatments for atherosclerosis is the use of statin medications. Recent studies have indicated that statins, in addition to their lipid-lowering effects, exert inhibitory and/or stimulatory effects on the NLRP3 inflammasome and TLRs. Some of the statins lead to activation of the inflammasome and subsequently cause secretion of IL-1β and IL-18. Thus, these actions may further aggravate the disease. On the other hand, some statins cause inhibition of the inflammasome or TLRs and along with lipid-lowering, help to improve the disease by reducing inflammation. In this article, we discuss these contradictory studies and the mechanisms of action of statins on the NLRP3 inflammasome and TLR pathways. The dose-dependent effects of statins on the NLRP3 complex are related to their chemistry, pharmacokinetic properties, and danger signals. Lipophilic statins have more pleiotropic effects on the NLRP3 complex in comparison to hydrophilic statins. Statins can suppress TLR4/MyD88/NF-ĸB signaling and cause an immune response shift to an anti-inflammatory response. Furthermore, statins inhibit the NF-ĸB pathway by decreasing the expression of TLRs 2 and 4. Statins are cost-effective drugs, which should have a continued future in the treatment of atherosclerosis due to both their immune-modulating and lipid-lowering effects.

## Introduction

Chronic inflammation has been recognized as an important factor in the development of inflammatory diseases such as atherosclerosis, which leads to functional and structural abnormalities of vascular walls. Atherosclerosis develops as a result of genetic and environmental interactions that cause inflammation and ultimately leads to endothelial cell dysfunction and thrombotic blockage. Elevated cholesterol and LDL-cholesterol (LDL-C) levels are the main risk factors that associated with the formation of atherosclerotic plaques and the development of atherosclerosis [1], which is characterized by lipid deposition, leukocyte infiltration, and smooth muscle cell proliferation in the vascular walls [2, 3].

Innate immunity is the first line of defense in the human body, which occurs in response to recognized antigens via receptors or so-called pattern-recognition receptors (PRRs). These receptors are found on innate immune cells such as macrophages, neutrophils, and other inflammatory cells [4]. The PRRs are divided into five groups: toll-like receptors (TLRs), NOD-like receptors (NLRs), RIG-I-like receptors (RLRs), C-type lectin receptors (CTLs), and AIM2-like receptors (ALRs). Some of them are located on the membrane of the cell (e.g., TLRs and CTLs), while other families are cytoplasmic receptors and expressed in the cytoplasmic space (e.g., NLRs and RLRs) [5]. The PRR-related signaling pathway has recently been identified as the bridge between the innate immune system and inflammatory diseases such as atherosclerosis and autoimmunity, especially NLRs and TLRs [6–8]. NOD-like receptors (NLRs), especially NLRP3, are the well-known receptor that detects the endogenous danger signal created by the presence of cholesterol crystals (CCs), which leads to activation of the inflammasome complex that activates caspase-1. Subsequently, active caspase-1 leads to proteolytic cleavage of pro-inflammatory cytokines such as pro-IL-1β and pro-IL-18 into their mature and active forms [9], which are the most important cytokines involved in the progression of atherosclerosis. The presence of CCs and elevated LDL are the main stimulators involved in the pathogenesis of atherosclerosis, which leads to NLRP3 inflammasome activation [10]. Moreover, the TLRs can identify endogenous ligands such as oxidized LDL (ox-LDL), heat shock proteins (HSP), high mobility group box 1 protein (HMGB1), fibrinogen, beta-defensin, and heparan sulfate. These endogenous proteins are often released in response to stress or tissue damage at sites of chronic inflammation [8]. Therefore, TLRs may be involved in pathological inflammatory diseases such as atherosclerosis.

Several anti-inflammatory drugs have been tested for their potential of reducing atherosclerosis risk. Statins are a good example of anti-inflammatory drugs that reduce the effects of inflammatory cytokines. Recent studies have shown that statins, in addition to their lipid-lowering effects, exert inhibitory or stimulatory effects on the NLRP3 inflammasome and TLRs. Some of the statins mediate an activation of the inflammasome, which subsequently leads to the secretion of IL-1β and IL-18. Thus, this action may result in further aggravation of the CVD. On the other hand, some statins cause inhibition of the inflammasome or TLRs, which leads to a reduction in inflammation. Together with their lipid-lowering effects, this outcome results in an improvement of the disease. In this review, we focus on these conflicting studies and the mechanisms of action of statins on the NLRP3 inflammasome and TLRs pathways.

## Inflammatory Responses and Atherosclerosis

### Start of Inflammation

Atherosclerosis is a complex inflammatory process characterized by fat deposition, leukocyte infiltration, and smooth muscle cell proliferation in the vascular walls [2, 3]. Since the atherosclerotic process occurs predominantly in the absence of microbial infection, it is considered a ‘sterile’ inflammation [11]. Chronic inflammation of the vessel walls is the main stimulator that is involved in the pathogenesis of atherosclerosis [10]. Additionally, macrophages are the most dominant cells involved in atherosclerosis [12]. For the first time, Ross R. et al. described a key event in the formation of atherosclerotic plaque, which included the role of macrophages in trans-endothelial migration, the proliferation of smooth muscle cells, the involvement of lymphocytes, and their migration to the intima [13]. Lipoproteins present in the vascular walls cause cholesterol crystallization, which is an aggravating factor in atherosclerosis and causes cellular damage and apoptosis. The CCs are hallmarks of advanced atherosclerotic plaques and indicate the instability and vulnerability of plaques to rupture [14].

### Progression of Inflammation

The accumulation of LDLs in the sub-endothelial environment is an essential event for the onset of atherosclerosis. Endothelial dysfunction occurs when endothelial cells are damaged or subjected to conditions of metabolic stress. One of the characteristics of endothelial dysfunction is the increased production of reactive oxygen species (ROS) [15]. ROS contribute to the oxidation of accumulated LDL and ultimately produce ox-LDL. Oxidized LDL exerts pro-atherogenic effects and it can induce the expansion of inflammatory macrophage phenotypes and lead to the formation of foam cells and endothelial dysfunction in the sub-endothelial site. Another way of generating these atherogenic species is via inflammatory cells, which by producing phospholipase A2 (PLA2), particularly lipoprotein-associated PLA2 and type II secretory PLA2, leads to additional oxidation of the phosphides in LDL particles and eventually the creation of atherogenic species [16]. Ultimately, ox-LDL activates endothelial cells, which subsequently produce adherent molecules such as vascular cell adhesion protein-1 (VCAM-1), intercellular adhesion molecule-1 (ICAM-1), and selectins, which are accompanied by complement factors and chemoattractant mediators such as IL-8 and monocyte chemoattractant protein-1 (MCP-1). Consequently, these molecules mediate the translocation of mononuclear cells to the intima. Also, macrophages and neutrophils participate in atherothrombosis. In some patients, atherothrombosis occurs following the rupture of an atheroma, which may be triggered through neutrophil extracellular traps (NETs). The NETs are networks of extracellular fibers, primarily composed of DNA from neutrophils, chromatin, serine proteases, and nuclear proteins, which bind pathogens. Plaque rupture triggers fibrin deposition and platelet aggregation at the initial site to entrap circulating red blood cells (RBCs). The interaction of thrombin-activated platelets with polymorphonuclear leukocytes (PMNs) at the site of plaque rupture during acute MI results in local formation of NETs and causes atherothrombosis.

### Stability of Inflammation

As discussed, intrinsic immune cells have receptors that are activated following the identification of pathogen-associated molecular patterns (PAMPs) and damage-associated molecular pattern molecules (DAMPs). Oxidized-LDL, as a DAMP molecule, is linked to CD36 and is phagocytosed by macrophages. However, CD36 is resistant to macrophage lysosomal enzymes, which leads to the accumulation of ox-LDL in macrophages and the formation of foam cells. This process ultimately produces a wide range of pro-inflammatory cytokines (e.g., IL-1, IL-8, IL-31, L-18, and TNF-α), as well as the expression of receptors (e.g., CD40L and CD40) [17]. Data from both experimental and epidemiological studies confirm a significant role of IL-6 and TNF-α, and especially IL-1β cytokines, in the development of atherosclerosis. [18].

**Fig. 1:**
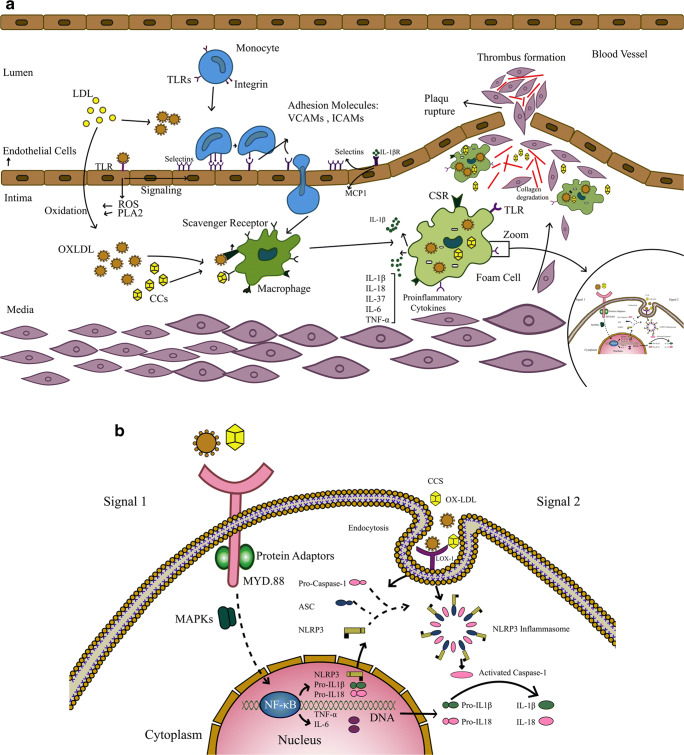
The roles of NLRPs and TLRs pathways in atherosclerosis pathophysiology. **a** At first, cholesterol and LDL deposit on vascular walls. Endothelial cells are stimulated through TLRs and resulted in ROS and PLA2 production. The ROS and PLA2 enzymes affected on accumulated LDL and cholesterol and produced ox-LDL and CCs. The ox-LDL and CCs ultimately, active endothelial cells and produce adherent molecules such as VCAM-1, which they evoke the monocular cells to the vascular wall. The ox-LDL and CCs as DAMPs are linked to NLRs and TLRs on phagocytized but they are resistant to macrophage lysosomal enzymes thus leads to the accumulation in macrophages and formats the foam cells which produce the wide range of pro-inflammatory cytokines. The IL-1β is one of the most important cytokines in atherosclerosis plaque and it has many roles such as increases the expression of adhesion molecules, which causes immune cells migration to the atherogenic site, smooth muscle cells proliferation, and MMPs activation, which lead to plaque rupture and obstructive thrombosis. **b** The stability of inflammation in atherosclerosis is required two signals; the initial signal is triggered via PRR receptors such as TLR, which activates NF-ĸB pathway that resulting in transcription of NLRP3 and pro-inflammatory cytokines such as pro-IL-1β, and the second signal is oligomerisation of activated NLRP3, and then call caspase-1 via Asc adaptor and eventually the formation of the inflammasome, which leads to proteolytic cleavage and activates pro-inflammatory cytokines such as pro-IL-1β and pro-IL-18 (abbreviations: CSR: cellular stress response is a macrophage scavenger receptor. IL-1βR: interleukin-1β receptor. LDL: low-density lipoprotein. OXLDL: oxidized LDL. NLRs: NOD-like receptor. NLRPs: Is an NLRs. TLR: toll-like receptor. VCAMs: vascular cell adhesion protein. ICAMs: intercellular adhesion molecule. MCP1: monocyte chemoattractant protein-1. ROS: reactive oxygen species. PLA2: phospholipases A2. CCs: cholesterol crystals. DAMPs: damage-associated molecular patterns. MMPs: matrix metalloproteinases. PRR: pattern recognition receptors. NF-ĸB: nuclear factor kappa-light-chain-enhancer of activated B cells. Asc: apoptosis-associated speck-like protein containing a CARD. MAPKs: mitogen-activated protein kinase. TNF-α: tumor necrosis factor-α. IL: interleukin. Lox-1: lectin-type oxidized LDL receptor-1. MyD-88: myeloid differentiation primary response-88)

Cholesterol crystals (CCs) and ox-LDL play a primary role in plaque formation and are associated with inflammasome activation. The inflammasome was discovered in 2002 and is composed of a complex of proteins that are involved in the maturation and secretion of IL-1β and IL-18 [9]. Recent studies suggest that the inflammasome plays a central role in the pathogenesis of cardiovascular diseases such as atherosclerosis, hypertension, and vascular inflammation [19]. It has also been shown that ox-LDL enhances the expression of TLRs. This is probably due to ox-LDL acting as a ligand for TLRs and subsequent signaling through the NF-ĸB transcription factor, which eventually leads to the synthesis of inflammatory cytokines and chemokines [20]. As shown in Fig. 1, the accumulation of CCs and the activation of PLA2 are the beginning of the inflammatory process and the migration of immune cells into the intima. This process eventually leads to activation of the inflammasome, TLRs, and the subsequent production of pro-inflammatory cytokines such as IL-1β and IL-18.

## The Role of IL-1β in Atherosclerosis Development

Cytokines are signaling molecules and regulatory agents of the immune system, which act as the main mediators of immune-modulation and inflammation. These molecules include diverse groups that clustered into several families such as the chemokines, interleukins (ILs), interferons (IFNs), tumor necrosis factors (TNFs), transforming growth factors (TGFs), and colony-stimulating factors (CSFs). These cytokines are categorized as having anti- or pro-atherogenic properties, which are expressed at atherogenic sites [21–23]. All immune and non-immune cells that may potentially play a role in the pathogenesis of CVD produce and react to these cytokines (Fig. 1).

The IL-1 family includes seven pro-inflammatory cytokines (e.g., IL-1α, IL-1β, IL-18, IL-33, IL-36α, IL-36β, and IL-36γ), antagonistic receptors (e.g., IL-1Ra, IL-36Ra, and IL-38), and an anti-inflammatory cytokine (e.g., IL-37), which have central roles in immune-based inflammation [24]. The family of IL-1 molecules is secreted from various cells such as macrophages, endothelial cells, and pancreatic islet cells. These inflammatory and intrinsic immune defense properties are referred to as ‘inherent immunity’ and enhance the response to acquired immunogenic antigens [25, 26]. Most immune cells express IL-1 family members or receptors, so most immune cells are affected by signaling mediated through the IL-1 family, such as the members IL-1α, IL-1β, and IL-18. Furthermore, this family of molecules plays an important role in the differentiation and function of lymphoid cells in acquired and inherent immune responses [24]. Inflammatory cytokines, particularly IL-1β and IL-18, play varying roles in the development of atherosclerosis [27]. IL-1α and IL-1β are the most well-known members of the IL-1 family and are referred to as precursors. Although the IL-1α precursor is biologically active, the IL-1β precursor requires proteolytic cleavage to the active form. Its activation is controlled and regulated at different levels [28]. Production and synthesis of IL-1β is monitored and controlled by complex mechanisms. Two signals are required for this process. Initially, the stimulation of pathogen recognition receptors (PRRs) by pro-inflammatory stimuli eventually leads to NF-ĸB induction. The second signal involves activating caspase-1 so that pro-IL-1β can be converted into the biologically-active form [29]. Caspase-1 is the main processor of IL-1β and is abundantly found in hematopoietic cells [9]. In addition to the caspase-1 pathway, other enzymes such as protease 3 (pr3) in neutrophils [30] and other proteases such as neutrophil elastase (NE) [31], matrix metallopeptidase-9 (MMP-9) [32], and cathepsin G, as well as kinase in mast cells [33], are all involved in the extracellular processing of IL-1β.

Many in vitro and in vivo studies have shown the role of IL-1 in atherosclerosis and CVD and have provided strong evidence that IL-1β is a potent pro-atherogenic factor [34, 35]. The increased levels of IL-1β observed in atherosclerotic lesions located in coronary arteries are associated with the severity of the disease [36]. The role of this cytokine has been widely investigated and documented for all stages of atherosclerosis development [37]. This pro-inflammatory mediator is involved in endothelial dysfunction and affects the arterial wall and endothelial cells, as well as increases the expression of adhesion molecules. Increased expression of adhesion molecules leads to the migration of immune cells to the atherogenic site; specifically, macrophages enter the intimal space during the initial phase. Macrophages, in turn, induce the secretion of inflammatory molecules such as chemokines and acute phase reactants such as IL-6, IL-8, MCP-1, and plasminogen activator inhibitor-1 (PAI-1) from endothelial and smooth muscle cells. These events increase the inflammatory macrophage phenotype, which exerts effects on cardiac muscle cells to promote their proliferation [19].

It has been shown that in IL-1 knockout mouse models of atherosclerosis, there is a decrease in foam cell formation and lesion size [38]. Moreover, IL-1 plays a significant role in the expression and activation of proteases such as MMPs, which ultimately lead to plaque rupture and obstructive thrombosis [39]. Thus, IL-1 plays a critical role in the onset, progression, development, and instability of plaque and clot formation in atherosclerosis. The expressions of IL-1α and IL-1β have also been reported during the development of atherosclerotic plaque. In fact, complex plaques have higher expressions of this cytokine in comparison to simple plaques, while healthy coronary arteries have the lowest expression of this cytokine [40]. Clinical studies have shown that the plasma levels of IL-1β are higher in CAD patients in comparison to non-CAD patients, and this finding is associated with an increased incidence of cardiovascular events in patients who have atherosclerotic risk factors [41–43]. In addition, various animal models have demonstrated the pro-atherogenic properties of the IL-1β cytokine. In this regard, silencing IL-1β in apoE-deficient mice (ApoE^−/−^) that are susceptible to atherosclerosis has revealed a reduction in the rate and extent of coronary atherosclerosis [35]. Experimental studies on atherosclerosis-susceptible animals have shown that either genetic deletion, or pharmacological inhibition of IL-1 signals, reduces both the formation and rate of progression of atherosclerotic plaques. In contrast, either an increase in active IL-1 that is formed after the injection of exogenous IL-1β, or a reduction in IL-1Ra, can lead to plaque enlargement and further the progression of atherosclerosis [44].

Based on these findings, the Canakinumab Anti-Inflammatory Thrombosis Outcomes Study (CANTOS trial) designed to show the importance of the IL-1 pathway in atherosclerosis. This study investigated the role of IL-1 in inflammation during atherosclerosis and the protective potential of anti-inflammatory drugs during CVD development. In this trial, canakinumab (a monoclonal antibody against IL-1β) was examined for its potential in reducing the number and frequency of secondary myocardial infarction (MI) events in cardiac patients. The results of this trial demonstrated that canakinumab, by decreasing inflammation, resulted in a reduced risk of cardiovascular events without affecting blood cholesterol levels. Therefore, it was the first large-scale randomized study that confirmed the hypothesis that IL-1β was pro-atherogenic and proved that inhibition of inflammation has a beneficial effect on the progression of CVD without an effect on fat lowering [45, 46]. On the other hand, interventional treatments with anakinra as a recombinant IL-1 receptor antagonist (IL-1Ra), rilonacept, and canakinumab suggested a significant pathological role of IL-1β in auto-inflammatory diseases [47]. Therefore, these studies provided evidence that IL-1β inhibition could improve the clinical outcome of CVD.

## NLRP3 Inflammasome and Its Role in Atherogenesis

**Fig. 2:**
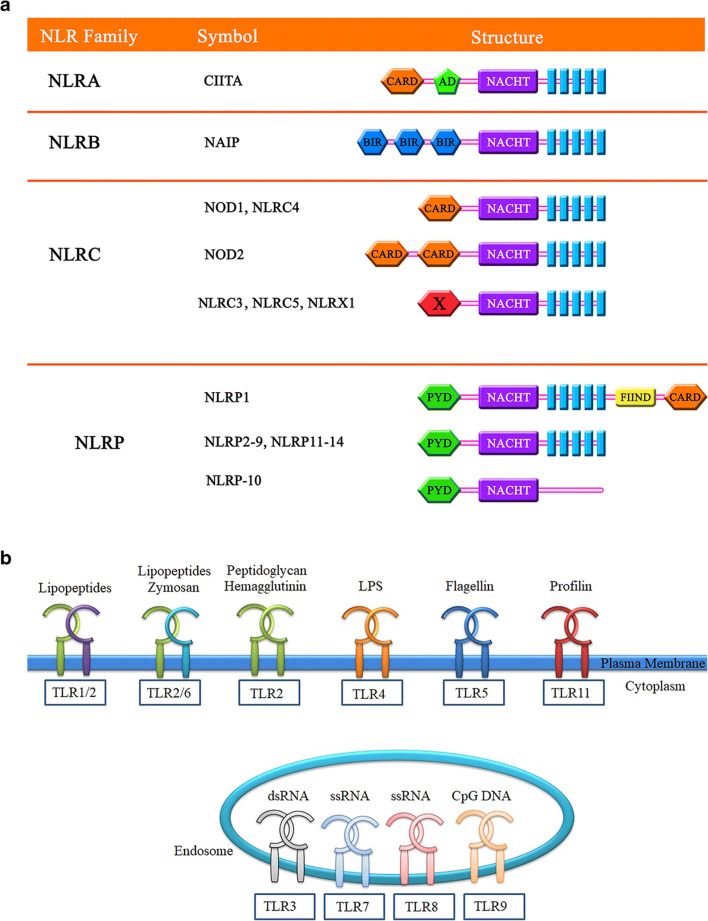
The NLRs and TLRs families’ structure. **a** The NLR families are divided into five sub-families including NLRA, NLRB, NLRC, NLRP, and NLRX. The symbols and structures of the 22 members are shown. **b** Cell surface and intracellular TLRs and their ligands. TLRs are divided into two groups based on their cellular localization. TLRs 1, 2, 4–6, and 11 localize to the cell surface and TLRs 3 and 7–9 reside at endosomal compartments. Cell membrane TLRs respond to microbial membrane antigens such as LPS, whereas endosomal TLRs recognize the virus and bacteria-derived nucleic acids (abbreviations: NLR: Nod-like receptor. CARD: caspase recruitment domain. PYD: pyrin domain. NACHT: nucleotide-binding and oligomerization domain. LRR: leucine-rich repeat. BIR: baculoviral inhibition of apoptosis protein repeat domain. NAD: NACHT-associated domain. AD: acidic transactivation domain. FIIND: domain with function to find. TLR: toll-like receptor. ssRNA: single-strand RNA. dsRNA: double-strand RNA. CpG DNA: CpG oligodeoxynucleotide. LPS: lipopolysaccharides)

NLRs composed of 22 members are classified into functional sub-groups based on their N-terminal region. The most well-known members of this family are NOD1, NOD2, and NLRP3, which primarily detect PAMPs and DAMPs ligands (Fig. 2). Unlike NOD1 and NOD2, most ligands of the NLRP3 are endogenous like DAMPs and signals that indicate host damage (e.g., metabolic stress, including cell necrosis, extracellular matrix components, and multiple host-derived molecules such as uric acid and cholesterol following their conversion to the crystalline state) [48]. The NLRP3 inflammasome plays a role in proteolytic processes and the maturation of IL-1β to its functional form. It functions as an essential sensor in the pathology associated with vascular endothelial dysfunction [49]. Recently, it was shown that excessive activation of NLRP3 represented a pivotal mechanism in the pathogenesis of metabolic diseases such as type 2 diabetes, obesity, gout, and atherosclerosis [50, 51]. In vitro experiments have demonstrated that cholesterol is the most important signal for NLRP3 activation [52]. As shown in Fig. 1, two independent signals are required for NLRP3 activation: the initial signal is triggered via PRR receptors such as TLR and IL-1R, which activate the NF-ĸB pathway and result in transcription of NLRP3 and pro-IL-1β. The second signal is oligomerization of activated NLRP3 and subsequently the recruitment of caspase-1 via ‘apoptosis-associated speck-like protein containing a caspase recruitment domain’ (Asc) adaptor. This process eventually causes the formation of the inflammasome, which ultimately leads to proteolytic cleavage and the activation of pro-inflammatory cytokines such as pro-IL-1β and pro-IL-18 [9].

Both the mRNA and protein levels of NLRP3 in peripheral blood mononuclear cells (PBMCs) obtained from CAD patients are higher than non-CAD controls, which suggest that the transcription and protein levels of the NLRP3 inflammasome have a significant positive correlation with elevated plasma levels of IL-1β and IL-18 [41]. The NLRP3 knockout gene in experimental high-fat diet animal models shows a reduced production of IL-1β and IL-18 [43]. Additionally, studies have also shown that atherosclerotic plaques have higher NLRP3 mRNA, Asc adaptor, caspase-1, IL-1β, and IL-18 levels [53]. In atherosclerotic plaques, components such as ox-LDL, CCs, extracellular ATP, and ROS may possibly be activating the NLRP3 inflammasome, which leads to inflammation and cellular infiltration [54, 55]. Recent studies have also determined that the P2X7 receptor (P2X7-R) that mediates ATP-dependent NLRP3 activation is significantly increased in coronary atherosclerotic lesions of ApoE^−/−^ and LDL-R^−/−^ deficient mice, as well as in human carotid atherosclerotic plaque tissue [56, 57]. Additionally, the P2X7-R knockdown in ApoE^−/−^ [56] and LDL-R^−/−^ deficient mice [57] showed a decrease in the activation of lesional inflammasomes and the size of atherosclerotic plaques. Thus, this would seem to suggest that P2X7R is involved in the progression of atherosclerosis by promoting the activation of the NLRP3 inflammasome [58, 59].

The first experimental study that demonstrated the importance of NLRP3 activation in the development of diet-induced atherosclerosis was performed by Duewell P. et al. [19]. They transplanted the bone marrow of wild-type, NLRP3^−/−^, Asc^−/−^, IL-1β^−/−^, IL-1α^−/−^ mice into LDL-R^−/−^ mice. After 8 weeks of consuming a high-fat diet, they found that the NLRP3^−/−^, Asc^−/−^, IL-1β^−/−^, and IL-1α^−/−^ mice showed a significant decrease in the number of atherosclerotic lesions. Their findings suggest that NLRP3, Asc, IL-1β, and IL-1α represent important inflammatory biomarkers in LDL-R^−/−^ mice when compared to wild-type mice. This study also revealed the importance of NLRP3 activation in the development of atherosclerosis. Another study employing a caspase-1 knockout in a high-fat diet animal model showed a significant reduction in the formation of atherosclerosis plaques. Furthermore, other studies have shown that silencing IL-1α, IL-1β, and caspase-1 genes in the ApoE^−/−^ mouse model can significantly decrease the development of atherosclerotic lesions [60].

Another study reported that bone marrow depletion of caspase-1 in LDL-R^−/−^ deficient rats consuming a high-fat diet had a significant reduction in atherosclerotic plaque formation, while other studies have shown that silencing IL-1α, IL-1β, and caspase-1 genes in ApoE^−/−^ mice can significantly diminish the development of atherosclerosis [60]. In this regard, Menu P. et al. reported contradictory results; ApoE^−/−^ mice with a double-knockout each of NLRP3^−/−^, Asc^−/−^, and caspase-1^−/−^ and consuming a high-fat diet for 11 weeks neither influenced the progression of atherosclerosis, nor the progression in cell infiltration into the intima. Therefore, this study showed that the progression of atherosclerosis was independent of the activation of NLRP3-mediated inflammation [61]. Baldrighi M. et al. showed that these different results could be explained by the difference in the test conditions including the mouse model employed, gender, diet type, and the duration of the experiment [62].

In in vitro studies using THP-1 cell lines, it has been demonstrated that NLRP3 gene silencing using siRNA caused a decrease in IL-1β secretion in response to CCs [62]. Zheng F. et al. found that knockout of the NLRP3 gene in ApoE-deficient mice using lentiviral RNA interference reduced the level of inflammatory cytokines, the lipid content of macrophages in atherosclerotic plaque, and increased the content of collagen in platelets. It also reduced the vulnerability of the plaques to rupture. Therefore, this study demonstrated that NLRP3 plays an important role in the pathogenesis of plaque vulnerability and instability [63]. All of these data demonstrate the critical role of the NLRP3 inflammasome in the pathogenesis of cardiovascular disease, especially atherosclerosis. Thus, targeting this molecule may very well represent a potential therapeutic intervention in the treatment of CVDs.

## Structure, Function, and Role of TLRs in Atherosclerosis

TLRs are part of the PRR family and identify unique patterns of microbial pathogens. These receptors are transmembrane proteins that possess a leucine-rich extracellular region and a conserved cytoplasmic region with homology to the IL-1 receptor, termed the Toll/IL-1 receptor homology region [64]. The TLR and IL-1 receptor members can potentially use the same signaling pathway [8]. TLR signaling stimulates the production of reactive nitrogen and oxygen species, anti-microbial peptides, inflammatory cytokines, adhesion molecules, and acute-phase proteins [64]. Thirteen distinct TLRs (TLR1–TLR13) have been identified in mammals. Some members of this family have been found in the plasma membrane (TLR-1, 2, 4, 5, 6, 10, and 11) while others are localized intracellularly (TLR-3, 7, 8, and 9) [65]. This distribution allows for the identification of a wide range of intracellular and cytoplasmic PAMPs (Fig. 2). Activation of TLR signaling can subsequently activate both innate and acquired immune responses through several mechanisms such as expression and release of inflammatory cytokines and anti-microbial peptides, dendritic cell (DCs) maturation, and up-regulation of co-stimulatory molecules on activated dendritic and T cells [66]. The TLRs can recognize endogenous ligands such as ox-LDL, HSPs, hyaluronan fragments, HMGB1, fibrinogen, beta-defensin, and heparan sulfate. These endogenous proteins are often released in response to stress or tissue damage at chronic inflammation sites. Therefore, due to the identification of self-ligands, TLRs may be involved in pathological inflammatory diseases such as atherosclerosis [8] (Fig. 1). Previous studies have shown up-regulation of TLR1, 2 and 4 in atherosclerotic plaques [64]. Specifically, previous studies have shown that ox-LDL can increase TLR4 expression without an effect on TLR2. This is probably due to ox-LDL acting as a ligand for TLR4 and eventually activating the NF-kΒ transcription factor, which leads to the synthesis of inflammatory cytokines and chemokines [20].

Previous studies have shown up-regulation of TLR1, 2, and 4 in atherosclerotic plaques [67]. In fact, it has been demonstrated that ox-LDL increase TLR4 expression without an effect on TLR2 expression. This is probably due to ox-LDL acting as a ligand for TLR4, and consequently signaling through the NF-kΒ transcription factor that leads to the synthesis of inflammatory cytokines and chemokines [20]. TLR4 signaling requires an adapter protein called MyD-88. MyD88-deficient mice exhibit a significant decrease in inflammatory cytokines and chemokines in the early stages of atherosclerosis [68]. Also, it has been observed that inhibition of TLR4 activation can reduce plaque size and macrophage infiltration into atherosclerosis lesions [68]. Further evidence supporting the roles of TLR2 and TLR4 in atherosclerosis is the fact that TLR2 and TLR4 agonists can stimulate atherosclerosis in various mouse models [67, 69]. However, the role of endosomal TLR signaling pathways in atherosclerosis is not completely clear, because both pro- and anti-atherogenic properties of endosomal TLRs have been reported. In this regard, epidemiological studies have shown that cardiovascular risk is increased in individuals with chronic or recurrent bacterial infections [70] and increased concentrations of plasma LPS [71]. Thus, either infection or an increase in plasma LPS resulted in either TLR2 and TLR4 up-regulation on monocytes [72] or responsiveness of leukocytes to TLR2 and/or TLR4 ligands [73]. Recent studies have demonstrated that TLR signal transduction in immune cells is a potential therapeutic target in atherosclerotic cardiovascular disease. This is because enzyme and inflammatory cytokine production are directly related to increased TLR expression via NF-ĸB [74]. Therefore, these findings suggest that TLRs could be involved in inflammatory processes underlying atherosclerosis and may represent useful therapeutic targets in the treatment of CAD.

TLR4 is expressed at low levels by endothelial cells in the normal vessel wall, but increases in atherosclerotic plaques. This up-regulated TLR4 is often found in the rupture-prone shoulder of the atherosclerotic plaques [75] (Fig. 1). It has been shown that TLR4 is increased in macrophages in lipid-rich atherosclerotic lesions. Also, ox-LDL leads to up-regulation of TLR4 [76]. For the first time, Michelsen et al. showed a direct correlation between TLR4 signaling and the formation of atherosclerotic plaque. They observed that atherosclerosis was decreased in a TLR4 knockout hypercholesterolemic mouse model [77]. On the other hand, it has been determined that there is a reduction in neointimal formation in the ‘early’ stages of atherosclerosis in the absence of TLR4 signaling [78]. TLR2 signaling has also been demonstrated to be involved in the development of atherosclerosis [67]. In this regard, it has been reported that activation of endogenous TLR2 in mice may play a central role in the regulation of vascular inflammation, as well as subsequent neointimal formation in damaged vessels [79].

Currently, it hoped that the attenuation of the TLR signaling pathway may alter the progression of CAD. The PRIM study showed inconsistent results with the hypothesis that TLR4 polymorphisms such as D299G could reduce the risk of coronary events. This study determined that the D299G TLR4 polymorphism was not associated with CAD risk or any inflammatory disease [80]. Moreover, Yang et al. showed that the D299G polymorphism is not associated with coronary artery stenosis [81]. The D299G polymorphism is located in the TLR4 region on chromosome 9 and correlated with diminished activity due to a defect in TLR4 signaling [82]. Kiechl et al. reported that the D299G polymorphism is associated with an increased risk of acute severe infection, but a decrease in atherosclerosis, carotid artery disease, and overall cardiovascular mortality [83]. Additionally, it has been shown that this polymorphism may affect lipid metabolism in addition to lowering pro-inflammatory status. Carriers with this polymorphism have a lower risk for cardiovascular events following statin therapy in comparison to non-carriers [84]. In contrast with its protective effects, it has also been shown that D299G polymorphism can increase the risk of MI in men. The results of a 3-year follow-up study by Labrum et al. showed no association between the polymorphism and the progression of intima-media thickness (IMT) [85]. Another study with young male and female Finns did not show an association between the D299G polymorphism and carotid artery IMT. However, this group reported an association between the polymorphism and carotid artery elasticity which may involve incomplete TLR4 signaling [86].

## Statins and Their Mechanisms of Action

### Mechanisms of Action in Atherosclerosis

**Fig. 3:**
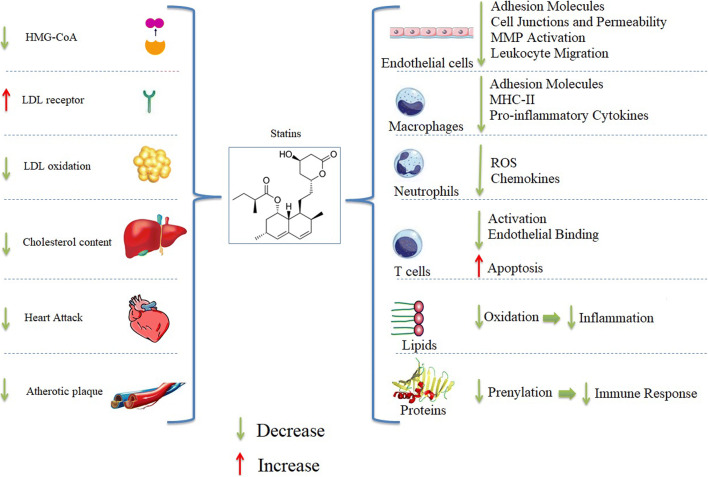
The effects of statins in atherosclerosis (**a**) and the immune system (**b**) (abbreviations: HMG-CoA: 3-hydroxy-3-methyl-glutaryl-coenzyme A. MMP: matrix metalloproteinases. ROS: reactive oxygen species. MHC-II: major histocompatibility complex-II. LDL: low-density lipoprotein)

Among a wide class of conventional and novel lipid-lowering therapies [87, 88], statins are the most commonly used effective drugs to treat hyperlipidemia in CVDs. These classes of drugs affect the mevalonate pathway through competitive inhibition of the rate-limiting enzyme in cholesterol neosynthesis called 3-hydroxy-3-methylglutaryl coenzyme A (HMG-CoA) reductase. HMG-CoA reductase converts mevalonate to cholesterol in the liver and other tissues and reduces reductase gene expression through negative feedback [89]. As a result, these drugs lead to a reduction in the cholesterol content in liver cells, and also cause increased expression of the LDL receptor on the cell surface that leads to a decrease in plasma LDL levels. Additionally, the normal functioning of the LDL receptors helps to maintain a normal concentration of intracellular cholesterol [90]. Moreover, statins can reduce plasma triglyceride levels without effect on the concentration of lipoprotein-A [91] (Fig. 3). Finally, several pleiotropic actions independent of their established lipid-lowering effects have been described for statins [92–96].

Hydrophilic statins such as pravastatin and fluvastatin do not easily penetrate into the cell membrane which results in less pleiotropic properties and fewer potential side effects for the central nervous system (CNS) and muscle. On the other hand, lipophilic statins such as simvastatin, lovastatin, and atorvastatin have more pleiotropic effects and side effects [94]. Overall, two generations of statins exist; natural statins (e.g., lovastatin, simvastatin, and pravastatin) and synthetic statins (e.g., fluvastatin, atorvastatin, and rosuvastatin), which have different pharmacokinetic properties, lipid-reducing properties, and complications with chronic use [97]. Although all of the statins reduce LDL, they have different effects on lowering cholesterol, the incidence of cardiovascular events, and the development of atherosclerosis. For example, pravastatin can reduce mortality in those who have had MI or experience unstable angina [98]. This difference may be due to either non-lipid agents, or non-cholesterol-dependent effects [99]. The most effective statin drugs in terms of reducing LDL and triglyceride levels are atorvastatin and rosuvastatin, respectively [100]. Higher doses of statins are more effective than lower doses for profound LDL reductions and eventually lead to reduced coronary artery diseases risk [101].

Statins are effective in preventing both primary and secondary cardiovascular disease. Large-scale randomized clinical trials showed beneficial effects of statins at reducing mortality in atherosclerosis patients, as well as a significant reduction of non-lethal vascular events [102, 103]. It has been suggested that the remarkable and significant beneficial effects may partly be due to the lowering of cholesterol levels, and other properties beyond cholesterol-lowering [104]. Since atherosclerosis has been identified as an inflammatory disease [105], extensive clinical studies have been performed and have shown potent anti-inflammatory effects of statins in the treatment of atherosclerosis [106]. Generally, the beneficial effects of statins in CVD are due to optimization of the lipid profile, inhibition of LDL oxidation, anti-inflammatory functions, inhibition of migrating immune cells, and inhibition of proliferation of smooth muscle cells. In addition, statins mediate anti-atherosclerotic properties by preventing the development of new lesions and stabilizing existing plaques via a reduction in the expression of tissue factor (TF). Moreover, statins indirectly cause an increase in endothelium-derived nitric oxide production and a reduction in platelet aggregation, which leads to a reduction in the risk of acute coronary events [107, 108].

### Mechanism of Actions in the Immune System

Beyond the role of reduced fats, statins have effects on the immune system. As shown in Fig. 3, their prominent effects include anti-inflammatory, anti-oxidant, anti-apoptotic, and are tissue-protective in some pathological conditions [109]. Statins affect the innate immune system through their effects on endothelial cells, macrophages, natural killer cells, and neutrophils, while they influence the acquired immune system by inhibiting antigen presentation and T cell polarization [110]. Evidently, the most predominant non-lipid-lowering effect of statins that has been reported includes their anti-inflammatory properties, which are multifaceted in nature. For example, some of these effects are due to the reduction of upstream mediators from cholesterol biosynthesis in the mevalonate pathway and, consequently, there is a reduction of protein prenylation that affects the immune response [110]. Decreased protein prenylation leads to a shift of the immune response to an anti-inflammatory response accompanied by a reduction in TNF and IL-6 levels in peripheral blood as seen in various LPS-treated animal models. It has been demonstrated that statins can reduce the activity of transcription factors such as NF-ĸB and activator protein-1 (AP-1), which are involved in regulating inflammatory pathways such as IL-1β [111]. Importantly, statins exert anti-inflammatory effects on several cells that are involved in the development and rupture of atherosclerotic plaques [112, 113].

Recent clinical trials have shown beneficial effects of statins in the reduction of inflammation. Statins reduce C-reactive protein (CRP) [114–116] as an acute-phase protein [117, 118]. Also, statins disrupt other inflammatory processes such as expression of cell adhesion molecules, inhibition of leukocyte migration to the sub-endothelial space, MHC-II down-regulation, and reduction in the CRP and TNF-α levels [114–116]. These observations suggest that statins can be used for other inflammatory conditions such as inflammatory arthritis [119, 120], diabetes [121], asthma [122], sepsis [123], and neurodegenerative diseases such as Alzheimer’s disease (AD) [124].

## The Effects of Statins on NLRP3 in CVD: Inhibitory or Stimulatory Effects

Several studies investigated statin effects on IL-1 and the NLRP3 inflammasome. It showed that statins such as simvastatin can reduce cytokine production in monocytes under TLR-stimulated conditions in patients with hyper-cholesterolemia [125, 126]. Statin therapy can down-regulate NLRP3, cathepsin-B, and downstream mediators such as IL-1β, which play a significant role in inflammation associated with atherosclerosis and reduce NLRP3 gene expression in PBMCs of CVD patients [59]. On the other hand, several studies demonstrated that statins can enhance IL-1β expression in response to LPS under the absence of any NLRP3 inflammasome stimuli [127, 128]. In addition, statins can enhance caspase-1 activity and promote IL-1β activation. This effect was initially reported to be associated with simvastatin and no other statins. Interestingly, this effect has now been reported to be associated with an increased risk of diabetes in some patients that have received statin therapy [129].

**Fig. 4:**
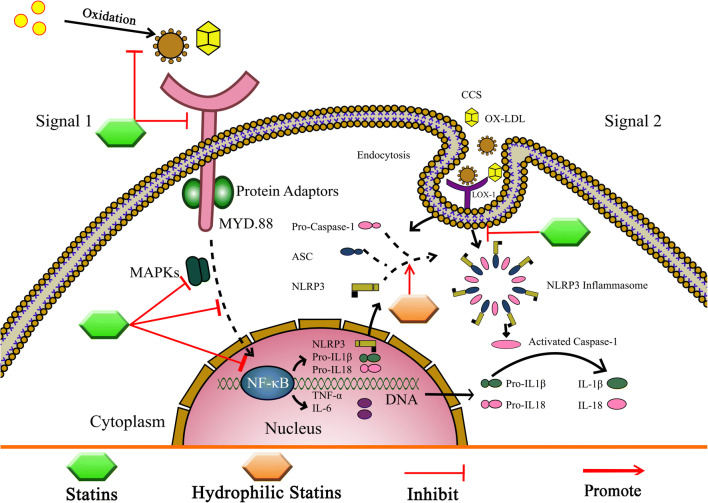
The effects of statin on NLRP3 inflammasome and TLRs pathways. It appears that the effects of statins on NLRP3 complex are related to chemistry and pharmacokinetics features. Lipophilic statins have more pleiotropic effects on NLRP3 complex compare than hydrophilic statins. In the other hand, statins with inhibition of the mevalonate pathway, the protein prenylation or lipidation, and decreases cholesterol production caused reduce TLR agonists and suppressing TLR4/MyD88/NF-ĸB pathway. Also, statins inhibit NF-ĸB through reduce MAPKs phosphorylation and stabilized IKB-α as an NF-ĸB inhibitor. Statins inhibit expression of LOX-1 receptor; therefore, they could inhibit the inflammation of NLRP3 through LOX-1/NF-ĸB pathway. The diabetes basic risk, metabolic syndrome, genetic, and lifestyle could promote NLRP3 inflammasome in presence statins. The statin could decrease the expression of TLRs 2 and 4, so suppressed NF-ĸB activity. At the genetic and molecular level, the TLR4 D299G polymorphism contributes in less susceptibility to cardiovascular disorders and increase response to statin therapy; also, statin therapy has a positive effect on miRNAs levels which affected on TLRs and negatively regulated TLR4 (abbreviations: LDL: low-density lipoprotein. OXLDL: oxidized LDL. NLRP3: Is a NLRs. CCs: cholesterol crystals. NF-ĸB: nuclear factor kappa-light-chain-enhancer of activated B cells. Asc: apoptosis-associated speck-like protein containing a CARD. MAPKs: mitogen-activated protein kinase. TNF-α: tumor necrosis factor-α. IL: interleukin. Lox-1: lectin-type oxidized LDL receptor-1. MyD-88: myeloid differentiation primary response-88)

The exact anti-inflammatory effects of statins are incompletely understood. Several studies showed that the anti-inflammatory effects of statins are exerted through the suppression of inflammasomes. Various investigators demonstrated that the effect of statin therapy on the NLRP3 inflammasome is varying and significantly dependent on the type of statin used. There are inconsistent data on the effects of statins on inflammasome and caspase-1 activation. Some studies suggest that statins activate inflammasomes and caspase-1 [129–135]. In contrast, other studies have reported that statins interfere with the activation of inflammasomes and caspase-1 [41, 59, 106, 136]. Therefore, NLRP3-related IL-1β expression through statins remains controversial and their exact mechanism(s) is unknown. In the next section, we discuss the primary effect(s) of statins on the NLRP3 inflammasome (Table 1 and Fig. 4).

**Table 1:** The effects of statin on NLRP3/IL-1β in CVD and other models

Statins	Animal model/cell line	Mechanism of actions	Main effect		Ref.
			Inflammatory	Anti-inflammatory	
Atorvastatin	-THP-1 cells	- Inhibiting NLRP3 inflammasome through suppressing TLR4/MyD88/NF-Kb		✔	[137]
	-THP-1 cells	- Diminish NLRP3 inflammasome levels		✔	[41]
	-Endothelium-derived cell line Ea.hy926 and smooth muscle cells (SMCs)	- Down-regulate the effects of NLRP3 inflammasome through inhibiting activation of NF-ĸB, AP-1 factors		✔	[138]
	- Vascular smooth muscle cells (VSMCs) and U937 mononuclear cells	- Inhibition of NLRP3 inflammasome through reduces NF-κB activation and chemokine expression		✔	[139]
	- Rabbit model of atherosclerosis and cultured VSMCs	- Decrease the expression of COX-2 mRNA that induced by IL-1β and TNF-α		✔	[140]
	-Human coronary artery SMCs	- Activated NLRP3 inflammasome through increased expression of NF-kB and AP1-mediated MMP-9	✔		[141]
	-Human LDL	- Electron donation and proton stabilization mechanisms lead to i inhibit lipid peroxidation in human LDL and phospholipid vesicles, and subsequently NLRP3 inflammasome inactivation may via		✔	[142]
Fluvastatin	- THP-1 cells	- Induced caspase-1 activation, increased ATP release, induced moderate ROS elevation and IL-1β production	✔		[131]
	-Human peripheral-blood mononuclear cells (PBMCs)	- Activation of caspase-1, secretion of IL-1β, IL-18 and IFNγ in Th1 cells.	✔		[143]
	- Rat	- Inhibit NF-ĸB through scavenging free oxygen radicals, and inhibited NADPH-dependent lipid peroxidation		✔	[144]
Lovastatin	- THP-1 cells	- Induced caspse-1 activation, increased ATP release, induced moderate ROS elevation and IL-1β production	✔		[131]
	-Human mesangial cells	- Inhibition of inflammation process through inhibiting NF-kB activation		✔	[145]
Pravastatin	- Rat	- Inhibit NF-ĸB through scavenging free oxygen radicals, and inhibited NADPH-dependent lipid peroxidation		✔	[144]
Rosuvastatin	- THP-1 cells	- No impact on NLRP3 inflammasome levels	-	-	[41]
	-Myocardial infarction (AMI) and unstable angina patients	- Activating inflammasome through down-regulating the expression of cathepsin-B and NLRP3.	✔		[59]
	--Endothelium-derived cell line Ea.hy926 and arterial SMCs	- Down-regulate the NLRP3 inflammasome effects through inhibiting activation of NF-kB, AP-1 factors.		✔	[138]
	-Mice	- Reduced VCAM-1, MCP-1 and MMP-9 expression in the vessel wall inhibited vascular expression of superoxide and p22^phox^ production and diminishing plasma 8-isoprostanes concentrations.		✔	[146]
Simvastatin	- Rat	- Negatively effects NLRP3 inflammasome through inhibition of the PI3K/Akt signal pathways		✔	[147]
	- PBMCs	- Significant reduction in the IL-1β levels in response to stimulation with CC.		✔	[148]
	-Bone Marrow-derived Macrophages and Cell Culture Mouse bone marrow-derived macrophages (BMDMs)	- Decrease inflammation through induction of an anti-inflammatory 28-KDa IL-1β isoform		✔	[106]
	- THP-1 cells	- Increase caspase-1 that leads to the processing of pro-IL-1 β	✔		[138]
	-endothelium-derived cell line Ea.hy926 and arterial SMCs	- Down-regulate the effects of NLRP3 inflammasome through inhibiting activation of NF-kB, AP-1 factors		✔	[127]
	-Normal human PBMCs and THP-1 cells	- Enhanced caspase-1 activity	✔		[128]
	- Human umbilical vein endothelial cells (HUVECs)	- Inhibition of Ox-LDL-mediated NLRP3 inflammasome		✔	[149]
	- HUVECs	- Migration of inflammasome cells to the endothelium through adhesion cytokine-mediated activation of the endothelium	✔		[150]
Mevastatin	- HUVECs	- Inhibition of Ox-LDL-mediated NLRP3 inflammasome		✔	[149]

Since macrophages and foam cells within atherosclerotic plaques predominantly express NLRP3 inflammasome components [151, 152], the role of NLRP3 inflammasome activation in the pathogenesis of atherosclerosis has been the primary focus. Kong F. et al. investigated the effect of atorvastatin on human THC-1 monocyte cell lines and reported that atorvastatin can inhibit NLRP3 expression, disrupt the activity of caspase-1, and eventually interfere with the secretion of IL-1β in these cells. This group suggested that the inhibitory mechanism of atorvastatin was through suppression of the TLR4/MyD88/NF-ĸB pathway [137]. These results support the effects of atorvastatin on NLRP3, and ultimately, on IL-1β expression, and suggest that atorvastatin may represent a promising agent in the treatment of atherosclerotic plaque. As mentioned, TLR4 plays an important role in initiating inflammation associated with atherosclerosis [153–156]. MyD88, as an important adaptor in the TLR4 signaling pathway, has subsequent regulatory effects on NF-kB [157].

NF-ĸB is an important factor that regulates the expression of many genes that are involved in inflammatory responses and effect atherosclerotic lesion development. Moreover, this factor influences the expression of genes involved in vascular diseases that could influence cell migration, promote inflammation, and the balance between cell proliferation and apoptosis [158, 159]. Interestingly, it has been suggested that blocking of NF-ĸB in vivo can prevent MI [160]. Statins inhibit NF-ĸB by scavenging free oxygen radicals and also stimulating the production of nitric oxide, which can subsequently induce and stabilize IKB-α as an NF-ĸB inhibitor [144, 161]. Studies have shown inconsistent results regarding the effects of statins on NF-ĸB. Some studies have demonstrated that atorvastatin reduces the activation of NF-ĸB in mononuclear cells and smooth muscle cells [139], as well as decreases NF-ĸB activity in experimentally induced atherosclerotic lesions in rabbits [140]. Moreover, it has also been shown that lovastatin reduces NF-ĸB activation in mesenchymal cells [145] and other statins, such as simvastatin, lead to an increase in NF-ĸB activation in endothelial cells [150]. In a study by Dichtl et al., it was demonstrated that statins such as simvastatin, atorvastatin, and lovastatin reduce the activation of hypoxia-inducible factor-1 (HIF-1), NF-ĸB, and AP-1 in vascular and endothelial muscle cells. Therefore, the use of these statin drugs for their anti-inflammatory and anti-proliferative effects has been encouraged for the treatment of atherosclerotic heart disease [138]. Further studies have supported the anti-inflammatory role of statins, such as with atorvastatin, which inhibits the NF-ĸB pathway in the human coronary artery [141] and in various experimental animal models [162].

A randomized clinical trial was conducted by Satoh M. et al. on 60 CAD patients who had no prior history of statin therapy and then assigned either of two statins (atorvastatin or rosuvastatin). The patients were followed for 8 months. They showed that NLRP3 inflammasome levels in CAD patients were higher than control subjects and there was a positive correlation between the increased levels of NLRP3 and IL-1β and IL-18 [41]. This study confirmed that atorvastatin, but not rosuvastatin, markedly decreased the expression and activation of the NLRP3 inflammasome, as well as the plasma levels of IL-1β and IL-18 in CAD patients. However, the in vitro studies with THP-1 cells showed that treatment with both atorvastatin and rosuvastatin affected NLRP3, IL-1β, and IL-18 supernatant levels in a dose-dependent manner. However, rosuvastatin had a less significant effect on IL-1β and inflammasome levels when compared to atorvastatin [41]. The reason for this difference is not clear, but Mason et al. showed that atorvastatin is more lipophilic than rosuvastatin and exhibits better cell penetration. Notably, it was shown that atorvastatin exhibits a powerful antioxidant effect in comparison to other statins such as pravastatin, rosuvastatin, and simvastatin [142]. However, another clinical study by Koksal et al. in diabetic patients with hyperlipidemia did not show any difference between atorvastatin and rosuvastatin in terms of attenuating oxidative stress [163]. The reason for this difference may be due to the dose of rosuvastatin that was used (2.5 mg/day versus 10 mg/day).

Various animal models have shown cardioprotective effects of rosuvastatin in myocardial infarction, autoimmune myocarditis and experimental hypertrophy [164–166]. The protective mechanisms of rosuvastatin against dilated cardiomyopathy (DCM) have not yet been fully elucidated. In a study by Luo et al. using a DCM rat model in the context of experimentally induced type 2 diabetes mellitus, 8 weeks of treatment with rosuvastatin resulted in a decrease in the levels of NLRP3, Asc adaptor, IL-1β, and mitogen-activated protein kinases (MAPKs) in cardiac tissue [136]. They observed that cardiac disorders caused by diabetes in the rosuvastatin-treated group were prevented, while this effect was minimal in down-regulated NLRP3 rats. Thus, it was suggested that rosuvastatin may exert a protective effect and decrease cardiovascular morbidity, which is dependent on inhibiting the NLRP3 inflammasome. Importantly, MAPKs are also critical signaling pathways in cardiac remodeling associated with DCM [167, 168]. In fact, several studies have suggested that enhanced phosphorylation of MAPKs can be normalized by rosuvastatin [169, 170], which has been suggested to be linked to the inhibition of MAPK. This is consistent with previous studies, which have suggested that an increase in the activation of ERK1/2, P38, and JNK may worsen the functional and structural performance of cardiomyocytes in diabetic rats. In a study by Luo et al., it was mentioned that treatment with rosuvastatin inhibited hyper-phosphorylation of MAPKs and could improve cardiac function. Therefore, rosuvastatin appears to be extremely beneficial, at least in diabetic rats, in improving cardiac performance [136].

Oxidized-LDL receptor 1 (LOX-1) is a lectin-like receptor and is a major ox-LDL receptor in epithelial cells. Moreover, it is a biomarker for many CVDs [171, 172] and increases in response to many atherogenic stimuli such as TNFα, ox-LDL, and free radicals [171]. Activated LOX-1 can lead to the production of ROS, mitochondrial DNA damage, and finally, induction in the expression of NLRP3 [173]. LOX-1 deletion in epithelial cells results in reduced NLRP3 activation through ox-LDL. Wang et al. suggested a new mechanism for the beneficial effects of statins as cardiovascular drugs. This group showed that statins (simvastatin, mevastatin, and atorvastatin) activated pregnane X receptors (PXRs) in epithelial cells. Silencing the PXR gene leads to elimination in the inhibitory effect of statins on NLRP3-mediated inflammation, whereas PXR agonists or excessive expression of PXR provides a similar effect to statins on NLRP3. For the first time, they showed that statins can inhibit the activation of NLRP3 inflammasomes in response to atherogenic stimuli such as ox-LDL and TNFα in epithelial cells via activation of the nuclear receptor PXR [149].

Following binding of ox-LDL to endothelial LOX-1, the NF-ĸB pathway is activated. Importantly, statins inhibit LOX-1 expression in a PXR-dependent state. Therefore, statins can inhibit the inflammation of the NLRP3 inflammasome in epithelial cells via the LOX1-/NF-ĸB pathway. These results show the important role of ox-LDL and LOX-1 in NLRP3 inflammasome activation. In addition, the effect of statin-activated PXR on the binding of NF-ĸB to the human NLRP3 promoter has been investigated, and the results showed that statin-activated PXR prevents NF-ĸB binding to the NLRP3 promoter in epithelial cells. In chromatin immunoprecipitation experiments, it has been shown that mevastatin inhibits the NF-ĸB binding to the promoter region of the human NLRP3 gene. Since NF-ĸB has a central role in regulating NLRP3 gene transcription, it follows that PXR inhibits the activation of NF-ĸB genes.

In a study by Lv et al., it was shown that simvastatin leads to inactivation of the NLRP3 inflammasome by inhibiting the secretion of HMGB1, as one of the major DAMPs that can activate the NLRP3 inflammasome. This reduction in HMGB1 secretion improves the functioning of tight junctions and vascular permeability. In fact, this study showed that simvastatin, by enhancing the tight junction–zonula occludens-1 (ZO-1) and VE-cadherin (VE-cad)-based adherens junctions in the endothelial cell layer, leads to an increase in permeability of the endothelial cell layer. Moreover, simvastatin can inhibit the NLRP3 inflammasome by influencing NADPH oxidase-dependent O_2_ production [174]. This result is consistent with other studies, which suggested that endogenous ROS participate in the formation and activation of the NLRP3 inflammasome [175]. This effect was accompanied by simvastatin’s inhibitory effect on the release of HMGB1, which leads to improved tight junctions in the vascular site and enhances our understanding of the anti-inflammatory effects of statins on vascular pathology beyond their lipid-lowering effects. In one study that investigated the protective effect of statins against ischemia-reperfusion (IR), the mechanism for the beneficial effects of statins was not completely elucidated. Han et al. investigated the effect of simvastatin on HMGB1 expression in myocardial ischemia. They demonstrated that simvastatin has a protective role in the heart against IR damage by reducing HMGB1 protein levels and attenuating the PI3K/Akt signaling pathway [147]. In other studies, it was shown that the 16673-34-0 molecule, an intermediate subunit in glyburide synthesis, leads to inhibition of both NLRP3 inflammasome formation in myocarditis and the infarct size resulting from myocardial ischemia, without affecting glucose metabolism [176]. Another study showed that NLRP3 was identified as an important target for ROS in terms of attenuating the inflammatory response in diabetic cardiomyopathy [53]. Therefore, it is believed that statins protect the heart against IR by inhibiting the NLRP3 inflammasome.

Several reports have shown that statins can increase the expression of IL-1 in response to LPS [127, 128]. However, the effect of statin therapy on NLRP3 activation by stimulants such as CCs was not investigated. Boland et al. examined the inhibitory effect of simvastatin on IL-1β expression and utilized CCs as a stimulator of NLRP3 in PBMCs of a cardiovascular animal model. These investigators found that statin therapy can enhance the activation and expression of IL-1β in response to NLRP3 stimulants and suggested that simvastatin exerted inhibitory effects on the NLRP3 inflammasome following stimulation of the NLRP3 pathway by CCs [148]. However, it would seem that additional studies are needed to investigate the underlying molecular mechanism(s) to more clearly elucidate the effects of simvastatin following activation of the NLRP3 pathway by CCs. The reported effects of statins on IL-1β are potentially controversial due to the existence of different IL-1β isoforms. Pro-IL-1 is a 31 kDa protein that is secreted as an active 17 kDa form by caspase-1 following inflammasome activation. Since currently used analytical methods in these studies cannot distinguish between IL-1β forms, it is unclear whether the secreted IL-1β form is the biologically active form. Davaro et al. suggested that statins suppress inflammation through an anti-inflammatory form of IL-1β. In fact, they mentioned a new signaling pathway following inflammasome activation by LPS ‘priming’, which resulted in the production of a 28 kDa IL-1β isoform that was independent of caspase-1. This 28 kDa isoform is an intermediate of the IL-1α isoform and is not biologically activated, but it does interfere with IL-1/IL-1R signaling [106].

Long-term treatment with ticagrelor, an antiplatelet medication, reduces cardiovascular events in patients with acute coronary syndrome [177, 178]. Ticagrelor, like statins, enhances the levels of extracellular adenosine, which increase the amount of prostacyclin and 15-Epi-lipoxin A4 [179, 180]. Certain clinical benefits may be related to the effects of adenosine, including the release of 15-Epi-lipoxin A4. In previous studies, the anti-inflammatory properties of ticagrelor were shown [181], but its effect on NLRP3-mediated inflammation has not been reported. A recent study by Birnbaum et al. found that ticagrelor reduced the up-regulation of NLRP3, Asc, IL-1β, IL-6, and caspase-1. This effect in combination therapy with rosuvastatin was significantly greater than either rosuvastatin or ticagrelor monotherapy [180]. This combination therapy also had a synergistic effect on inhibiting NLRP3 post-infarction and reducing myocardial infarction in an experimental rat model [182]. Recently, adenosine-related cardioprotective effects have also been confirmed in a large-species animal model (pigs) [183]. Yume et al. indicated that long-term use of ticagrelor alone decreased the serum level of IL-1β, IL-6, and TNF-α, while the combination with rosuvastatin leads to a significant reduction in IL-1β, IL-6, TNF-α, and CRP levels [184].

## The Effect of Statins on TLR4 in Atherosclerosis

It has long been thought that targeting TLRs to limit inflammatory disease might be a safe strategy. However, long-term use of TLR inhibitors may increase the risk of infection. Genetic evidence has suggested that TLR signaling, especially through either TLR2 or TLR4, is likely to alter the risk of CAD in humans [185]. Various studies have been conducted to determine the effect of statins on TLRs and downstream signaling in CAD. In this regard, statin therapy has been shown to decrease the expression of TLR4 on immune cells, decrease monocyte inflammatory responses to LPS via down-regulation of TLR4, and suppress NF-ĸB activity, which caused a decrease in the secretion of inflammatory cytokines [139, 186]. Additionally, statins, by inhibiting TLR4, can prevent LPS-induced activation of mononuclear cells and endothelial cells [187], decrease membrane expression of TLR4 and TLR2, and subsequently, inhibit the activity of LPS-activated monocytes in hyper-cholesterolemic patients [126] (Table 2 and Fig. 4).

**Table 2:** The effects of statin on TLRs in CVD other models

Statins	Animal model/cell line	Mechanism of actions	Main effect		Ref.
			Inflammatory	Anti-inflammatory	
Atorvastatin	-Murine pro-B cell lines	- Decrease NF-kΒ activation and inhibit TLR4 by a defect in the TLR4 recruitment to lipid raft		✔	[188]
	- Rabbit	- Inhibiting the TLR4 expression and P65 NF-kB in the TLR4/NF-kΒ signaling pathway		✔	[189]
	- THP-1 cells	- Inhibits both TLR4 mRNA and protein, therefore, decreased of TNF-α, IL-6 and IL-1β levels		✔	[190]
	-Human Monocytes	-Significantly decreased of TLR4 expression in CD14+ monocytes through the inhibition of protein prenylation, which decreases the LPS-stimulated TNF-α and IL-6 synthesis.		✔	[191]
	- Murine pro-B cell	- Inhibition of TLR4 and the MYD88-dependent pathway in a dose- and time-dependent manner		✔	[192]
	- Rats	- Down-regulate TLR4 mRNA expression via two different signaling networks; IKB-α stabilization and inactivation of ERK phosphorylation that inhibit NF-ĸB activation directly and indirectly, respectively.		✔	[193]
	- CAD Patients	- Decreases TLR4/MyD-88 and subsequently inhibits intrinsic immune response and oxidative stress		✔	[194]
	- CAD Patients	- Decreased miRNA-146a/b, IRAK1 mRNA, TRAF6 mRNA and TLR4 mRNA levels		✔	[194]
	- CAD Patients	- Up-regulation of miRNA let-7i expression in monocytes, and consequently down-regulation of the TLR4 signaling pathway		✔	[195]
	- CAD Patients	- Increasing in circulating TLR4-responsive miRNAs including miR-31, miR-181a, miR-16 and miR-145 induce down-regulation of TLR4 activity.		✔	[196]
Simvastatin	-Hypercholesterolemic patients	- Decrease TLR4 and TLR 2 membrane expression on monocytes and reduced IL-6 and IL-1β cytokines production		✔	[126]
	-Healthy male volunteers	- Reduce TLR4 and TLR2 expression in monocytes and decrease TNF-α and MCP-1 production.		✔	[197]
	-Hypercholesterolemic patients	-A high simvastatin dose or the combination of low-dose simvastatin with ezetimibe reduces to a similar extent TLR2, TLR4 membrane expression and LPS-induced IL-6 and IL-1β production		✔	[126]
Atorvastatin and/or simvastatin	- Human peripheral blood monocytes	-Suppressed the DC maturation and limited their production of tumor necrosis factor-α, IL-1β and IL-6. - Inhibited Th1 and/or Th17 polarization by downregulation of transcriptional factors T-bet and RORct expression. -Induced T regulatory cells with IL-10 production. -Disrupt T cell activation through regulating miRNA let-7c in dendritic cells.		✔	[198]
Fluvastatin	-Hyperlipidemic rabbit model	-Reducing TLR expression and significantly inhibits this process		✔	[199]
	-CHF patients	-Reduce TLR4 and TLR2 proliferation.		✔	[200]
	-CHF patients	-Suppressing the TLR signaling network in monocyte.		✔	[200]
	-Rat	- Inhibits TLR4 overexpression and subsequently TNF-α and NF-ĸB		✔	[201]

The effects of co-administration of atorvastatin and mevalonate in an LPS-stimulated mouse model resulted in a decrease in NF-ĸB activation and inhibition of TLR4. The underlying mechanism used to explain this effect was a defect in the recruitment of TLR4 to the lipid raft [188]. Additionally, atorvastatin exerts effects on the atherosclerotic plaque by controlling lipid regulation and inhibiting TLR4 expression, as well as affecting P65 NF-ĸB in the TLR4/NF-kΒ signaling pathway [189]. Yang et al. showed that atorvastatin (ATV) can inhibit TLR4 protein levels, as well as inhibit TLR4 at the level of gene transcription. As a result, atorvastatin decreases TNF-α, IL-6, and IL-1β levels in LPS-stimulated THP-1 monocytes [190]. Following approximately 4 weeks of atorvastatin administration, TLR4 expression was significantly reduced in CD14^+^ monocytes in vivo and in vitro in a dose-dependent manner. Its mechanism of action is thought to involve the inhibition of protein prenylation, which decreases the synthesis of LPS-stimulated TNF-α and IL-6 [191].

MyD-88 is one of the most important players in the TLR4 signaling pathway, which participates in the pathophysiology of myocardial injury [202]. It has been shown that the protective effects of atorvastatin on cardiomyocytes result from a decrease in TLR4/MyD-88 and subsequent inhibition of the intrinsic immune response and reduction in oxidative stress in injured rat myocardium [193]. In other studies, it has been shown that atorvastatin has an anti-inflammatory effect on atherosclerosis through TLR4 and the MYD88-dependent pathway in a dose- and time-dependent manner [192]. Wang et al. showed that atorvastatin down-regulated TLR4 mRNA expression through two different signaling networks; IKB-α stabilization that directly inhibits NF-ĸB activation and a second pathway that involves inactivation of ERK phosphorylation, which indirectly inhibits NF-ĸB activation [203]. High-dose administration of simvastatin has been demonstrated to decrease TLR4 and TLR2 membrane expression on monocytes and subsequently reduce the production of IL-6 and IL-1β cytokines [126]. Moreover, the statins, via TLR4, can mediate the stabilization of atherosclerotic plaques. Use of statins leads to TLR4 down-regulation on macrophages and endothelial cells in carotid atherosclerotic plaques, which is associated with a reduced risk of cerebrovascular ischemic events [204]. For the first time, Kapelouzou et al. have shown that the expression levels for TLR2, 3, 4, and 8 were significantly increased and correlated with the development of atherosclerosis in an experimental hypercholesterolemic rabbit model. Fluvastatin significantly inhibited this process and decreased inflammation by reducing TLR expression, which was strongly associated with an alteration in plaque regression and a decrease in the levels of various mediators involved with atherosclerosis [199].

Chronic heart failure (CHF) is a disorder that primarily affects the cardiovascular system. It has been shown that there is an increase in TLR4 and cytokines in the heart tissue of patients suffering from CHF [205, 206], whereas TLR2 is increased in myocardial dysfunction in mouse models [207]. TLR2 and 4 are highly-expressed in CHF and increased TLR4 expression is an essential feature in the progression of CHF. In a study by Földes et al., it was shown that TLR4 and TLR2 were increased in CHF patients and that fluvastatin reduced the levels of TLR4 and TLR2 in monocytes ex vivo over 24 h. Based on their findings, fluvastatin suppressed the monocyte TLR-signaling network and restrained the innate immune response in CHF patients [200]. Therefore, the use of an appropriate dose of fluvastatin in patients would presumably decrease myocardial TLR4 expression and could potentially exert a cardioprotective role by attenuating the inflammatory response. Importantly, fluvastatin has been shown to significantly reduce MI/RI by inhibiting TLR4 overexpression and subsequently reduce TNF-α and NF-ĸB [201]. Furthermore, simvastatin administration in high doses for 4 days was shown to reduce the expression of TLR4 and TLR2 in monocytes and decrease TNF-α and MCP-1 levels [197]. This effect is significant, because TNF-α and MCP-1 are essential for the progression of atherosclerosis [208, 209].

TLR4 polymorphisms may affect and modulate the effectiveness of statin therapy. In this regard, it has been shown that TLR4 and statin (either simvastatin or pravastatin) exerted a synergistic inhibitory effect not only on LPS-induced NF-ĸB activation, but also on the expression of IL-6 and TNFα. Therefore, this may represent an explanation concerning genetic epidemiological studies, which have suggested that the beneficial effect of statins for cardiovascular risk is dependent on the TLR genotype [210]. Among symptomatic men with coronary artery disease, this TLR4 variant modifies the efficacy of pravastatin in preventing cardiovascular events [84]. In a study by John et al., it was shown that patients who were carriers of D299G genotypes had less susceptibility to myocardial infarction when receiving statin treatment. These findings suggested that TLR4 gene variations contribute to inter-patient variation in susceptibility to coronary ischemic events, as well as the TLR4 genotype and statin therapy may have synergistic effects [75] (Table 2 and Fig. 4).

Notably, miRNAs can also regulate the effect of statins on the TLR4 pathway. The miRNA let-7i targets TLR4 mRNA and has a negative effect on the expression of this receptor that regulates the innate immune response in mammals [28]. It has been shown that miRNA let-7i levels in monocytes of CVD patients are decreased in comparison to non-CVD patients and results in increased TLR4 mRNA levels in the CVD patients. Thus, this finding suggested that down-regulation of let-7i is probably associated with overexpression of TLR4 in CVD patients [195]. Interestingly, the use of atorvastatin had a positive effect on miRNA let-7i levels in patients with CVD. However, rosuvastatin did not affect either miRNA let-7i or TLR4 levels. It appears that the beneficial effects of atorvastatin resulted from its lipophilic properties [195]. Additionally, it has been reported that plasma levels of circulating TLR4-responsive miRNAs including miR-31, miR-181a, miR-16, and miR-145 were decreased in CAD patients, which may suggest their involvement in the pathogenesis of atherosclerosis. Thus, combination therapy with a statin and RAS blockade (i.e., using either an ARB or ACEI) to increase circulating TLR4-responsive miRNAs would potentially induce down-regulation of TLR4 activity in CAD patients [196].

## Other Mechanisms of Statins on Inflammatory Pathways

Another mechanism of action of statins on activating immune cells is by abolishing TGF-β signaling in T cells, which leads to a decrease in plaque size [211]. Importantly, dendritic cells are involved in various stages of atherosclerosis as specialized antigen-presenting cells [212]. It is also known that ox-LDL stimulates dendritic and T cells to a pro-inflammatory phenotype [213]. Activated T cells via ox-LDL are essential for plaque rupture [214]. Oxidized-LDL can activate T cells through the influence dendritic cells and this process is inhibited by atorvastatin and rosuvastatin. In a study by Frostegård et al. it was shown that statins can disrupt T cell activation by interfering with the regulation of miRNAs in dendritic cells. These investigators showed that atorvastatin inhibits the induction of miRNA let-7c by ox-LDL in dendritic cells. Therefore, let-7c plays a major role in T cell activation via ox-LDL-stimulated dendritic cells [198]. It has also been reported that miRNA-146a is responsible for creating inflammatory conditions and is known to be dependent on NF-ĸB [215]. In fact, miRNA-146a exerts a regulatory effect on NF-ĸB-dependent genes including IRAK1 and TRAF6 by using a negative-feedback loop [215]. The role of miRNA-146 in TLR4 modulation and downstream cytokine signaling has been demonstrated in human monocytes [215] and it participates in anti-atherogenic effects independent of the lipid-lowering effects of statins. In this regard, it has previously been reported that patients with CVD not only have higher levels of miRNA-146a/b and its target genes (i.e., IRAK1 and TRAF6), but also increased levels of TLR4 in comparison to non-CVD patients [194]. Combination therapy with statins and RAS inhibitors (i.e., either and ARB, or ACEI) also resulted in decreased levels of miRNA-146a/b, IRAK1 mRNA, TRAF6 mRNA, and TLR4 mRNA in the monocytes of CVD patients, which could potentially explain the anti-atherogenic effects of statins [194]. It would appear that the mechanisms of action of statins as it pertains to TLR inhibition and downstream signaling include a reduction or defect in the TLR receptor, modulatory effects on NF-ĸB transcription factor, influence on adhesion-signaling molecules and proteins (including MYD88), reduction of IRAK1 and TRAF6 by miRNAs, and finally, disruption of T cell activation via miRNA let-7c in dendritic cells.

## Conclusion

One of the causal risk factors for CVD includes the deposition of cholesterol and LDL on vascular walls and subsequent stimulation of NLRs and TLRs that mediates the production of pro-inflammatory cytokines to stabilize inflammation. Inflammation results in the migration of immune cells to the atherosclerotic site, smooth muscle cell proliferation, and activation of MMPs, which ultimately leads to plaque rupture and obstructive thrombosis. Statins are one of the most effective treatments for slowing and/or halting the development of atherosclerosis. In addition to their lipid-lowering effects, statins exert inhibitory and/or stimulatory effects on the NLRP3 inflammasome and TLRs. The effects of statins on the NLRP3 complex and TLR pathways are related to their chemistry and pharmacokinetics properties, as well as ‘danger signals’ such as CCs and/or ox-LDL. Lipophilic statins have a greater number of pleiotropic effects on the NLRP3 complex in comparison to hydrophilic statins. In the context of atherosclerosis and, as mentioned above, CCs and ox-LDL act as ‘danger signals’ and cause a reduction in the NLRP3 inflammasome in the presence of statins, while metabolic and diabetic disorders cause an activation of the NLRP3 inflammasome. On the other hand, statins decrease cholesterol production through inhibition of the mevalonate pathway, as well as protein prenylation. The decrease in cholesterol production and protein prenylation primarily results in suppression of the TLR4/MyD88/NF-ĸB signaling pathway and a reduction in the formation of the NLRP3 complex. As a result, immune responses are shifted to an anti-inflammatory response.

Furthermore, statins inhibit NF-ĸB by decreasing the expression of TLRs 2 and 4, reducing the levels of LOX-1 receptors, exerting positive effects on inhibitory miRNAs, reducing protein adaptors, and stabilizing NF-ĸB inhibitors. This molecular signaling cascade ultimately has the effect of reducing the expression of TLRs and suppressing the activity of NF-ĸB.

In conclusion, statins are cost-effective drugs, which should have a continued future in the treatment of atherosclerosis due to both their immune-modulating and lipid-lowering effects. Inclusion of statin drugs into a pharmacotherapeutic regimen of medications aimed at combatting atherosclerosis [e.g., medications for inhibiting the absorption of intestinal cholesterol (ezetimibe), fibrates to lower serum triglycerides, antiplatelet drugs to prevent the formation of blood clots, newer proprotein convertase subtilisin kexin type 9 (PCSK9) inhibitors, etc.] should increase the pharmacological effectiveness of this approach and improve long-term outcomes in patients with CVD. In our opinion, the addition of statin drugs to an already existing regimen of anti-atherosclerotic medications should pose no problems with current clinical practice guidelines, as this class of lipid-lowering agents is typically already utilized, but should preferably be firmly incorporated into standard practice algorithms for the treatment of atherosclerosis in an effort to fully exploit the additional beneficial immune-modulating effects of statins.

### Confirmation of Figure Originality

The authors confirm the figures originality and they have not previously been published. All figures were drawn with ChemDraw, Microsoft office PowerPoint and Adobe Photoshop softwares.
